# Improved Cardiac MRI Volume Measurements in Patients with Tetralogy of Fallot by Independent End-Systolic and End-Diastolic Phase Selection

**DOI:** 10.1371/journal.pone.0055462

**Published:** 2013-01-31

**Authors:** Hendrik G. Freling, Petronella G. Pieper, Karin M. Vermeulen, Jeroen M. van Swieten, Paul E. Sijens, Dirk J. van Veldhuisen, Tineke P. Willems

**Affiliations:** 1 Department of Radiology, University of Groningen and University Medical Center Groningen, Groningen, The Netherlands; 2 Department of Cardiology, University of Groningen and University Medical Center Groningen, Groningen, The Netherlands; 3 Department of Epidemiology, University of Groningen and University Medical Center Groningen, Groningen, The Netherlands; University Hospital Düsseldorf, Germany

## Abstract

**Objectives:**

To investigate to what extent cardiac MRI derived measurements of right ventricular (RV) volumes using the left ventricular (LV) end-systolic and end-diastolic frame misrepresent RV end-systolic and end-diastolic volumes in patients with tetralogy of Fallot (ToF) and a right bundle branch block.

**Methods:**

Sixty-five cardiac MRI scans of patients with ToF and a right bundle branch block, and 50 cardiac MRI scans of control subjects were analyzed. RV volumes and function using the end-systolic and end-diastolic frame of the RV were compared to using the end-systolic and end-diastolic frame of the LV.

**Results:**

Timing of the RV end-systolic frame was delayed compared to the LV end-systolic frame in 94% of patients with ToF and in 50% of control subjects. RV end-systolic volume using the RV end-systolic instead of LV end-systolic frame was smaller in ToF (median −3.3 ml/m^2^, interquartile range −1.9 to −5.6 ml/m^2^; p<0.001) and close to unchanged in control subjects. Using the RV instead of LV end-systolic and end-diastolic frame hardly affected RV end-diastolic volumes in both groups and ejection fraction in control subjects (54±4%, both methods), while increasing ejection fraction from 45±7% to 48±7% for patients with ToF (p<0.001). QRS duration correlated positively with the changes in the RV end-systolic volume (p<0.001) and RV ejection fraction obtained in ToF patients when using the RV instead of the LV end-systolic and end-diastolic frame (p = 0.004).

**Conclusion:**

For clinical decision making in ToF patients RV volumes derived from cardiac MRI should be measured in the end-systolic frame of the RV instead of the LV.

## Introduction

Evaluation of right ventricular (RV) volumes and function is crucial in the management of patients with congenital heart disease [1], [2]. RV dysfunction is particularly a problem in patients with tetralogy of Fallot (ToF) due to longstanding massive pulmonary regurgitation. Irreversible RV dysfunction can be prevented by pulmonary valve replacement before a certain threshold value for RV end-systolic and end-diastolic volume is reached [3]–[7]. Cardiac magnetic resonance (CMR) imaging is the golden standard in the evaluation of RV volume and function, and plays an important role in the decision for pulmonary valve replacement in patients with ToF and pulmonary regurgitation [1]–[7].

To acquire accurate CMR derived volume measurements, correct selection of the RV end-systolic and end-diastolic frame may be important. In normal hearts, contraction of the RV lags slightly behind that of the left ventricle (LV) [8]. Most patients with ToF have a right bundle branch block (RBBB) which leads to intra- and interventricular dyssynchrony. This dyssynchrony significantly extends duration of RV contraction and delays timing of RV end-systole compared to the LV [7], [9], [10]. Additionally, timing of RV ejection and end-diastole may be delayed in patients with ToF [11], [12]. In many centers the RV end-systolic and end-diastolic frame is selected independently from the LV end-systolic and end-diastolic frame [13], [14]. However, the magnitude of the overestimation of RV end-systolic volume and underestimation of RV end-diastolic volume and ejection fraction is unknown. Therefore, others state that independent selection of the RV frame is unnecessary as the magnitude of the misrepresentation of RV volumes and function is too small to be of clinical importance.

The present study is the first to quantitatively document the influence of independent selection of the end-systolic and end-diastolic frame for the RV and LV, on RV volume measurements in a large group of patients with ToF and control subjects.

## Materials and Methods

### Study population

Our institution’s CMR database was searched to collect 65 of the most recent CMR scans of patients with ToF and 50 normal CMR scans performed in patients suspected for myocardial infarction (control subjects). Normal CMR scans were defined as normal anatomy, normal LV and RV contraction, normal LV and RV volumes and ejection fraction with no signs of infarction, and no valvular dysfunction [15]. Ischemia was ruled out by stress testing. In all patients electrocardiograms performed within 6 months to the CMR date were collected to evaluate rhythm and conductance disturbances. A RBBB was present when the longest manually measured QRS duration≥100 ms in combination with a terminal R wave in lead V1 and V2, wide S wave in I and V6 on the electrocardiogram [16].

RBBB is defined as complete when the QRS duration≥120 ms and defined as incomplete when the QRS duration is ≥100 ms and <120 ms [16]. CMR scans of patients with ToF were included when RBBB was the only conductance delay and no additional conductance delays were present. CMR scans of control subjects were excluded when conductance delays were present on the electrocardiogram [16].

This retrospective study was approved by the University Medical Center Groningen review board. Informed consent was not required according to the Dutch Medical Research Involving Human Subjects act.

### Cardiac magnetic resonance imaging

All subjects were examined on a 1.5-Tesla MRI system (Siemens Magnetom Sonata, Erlangen, Germany or Siemens Magnetom Avanto, Erlangen, Germany) using a 2×6 channel body-coil. After single-shot localizer images, for function analysis short axis cine loop images with breath holding in expiration were acquired using a retrospectively gated balanced steady state free precession sequence. Short axis slices were planned in end-diastole from two slices above the mitral valve plane to the apex. The following parameters were used: TR 2.7 ms, TE 1.1 ms, flip angle 80°, field of view 320 mm, matrix 192×192 mm, 25 frames per cycle, slice thickness 6 mm, interslice gap 4 mm, voxel size 1.7×1.7×6 mm.

Image analysis was performed using commercially available software (QMass version 7.2., Medis, Leiden, The Netherlands). The end-systolic and end-diastolic frame was defined as the frame with the smallest and largest volume, respectively.These frames were selected by visual assessment independently for the LV and RV. LV and RV contours were drawn manually by tracing the endocardial borders in every slice in the end-systolic and end-diastolic frame of the LV. Contour tracing was aided by reviewing the multiple phase scans in the movie mode. The papillary muscle and trabeculae were considered part of the cavum. Additionally, RV contours were drawn in the end-systolic and end-diastolic frame of the RV.

The basal slice was selected with aid of long-axis cine view images. The basal slice of the LV was defined as the most basal slice surrounded for at least 50% by the LV myocardium. When the pulmonary valve was visible in the RV basal slice, only the portion of the right ventricular outflow tract below the level of the pulmonary valve was included. For the inflow part of the RV, the blood volume was included when the ventricle wall was trabeculated and thick compared to the right atrium wall [15].

Stroke volume was defined as end-diastolic volume minus end-systolic volume. Ejection fraction was defined as stroke volume divided by end-diastolic volume.

### Reproducibility

RV contours were first drawn in the visually selected end-systolic and end-diastolic frame of the RV. To minimize intraobserver variability, RV contours drawn in the end-systolic and end-diastolic frame of the RV were copied to the LV end-systolic and end-diastolic frame and then adjusted to this frame.

To obtain intra- and interobserver reproducibility, contours were drawn independently twice by the first observer and once by the second observer in 25 scans of patients with ToF and 25 scans of control subjects. The end-systolic and end-diastolic frame was selected independently twice by the first observer and once by the second observer. There were at least two weeks between repeated contour drawing by the first observer. Both observers had more than 2 years experience with RV contour drawing.

### Statistical analyses

Descriptive statistics were calculated for all measurements as mean and standard deviation for normally distributed continuous variables, median with interquartile range (IQR) for skewed continuous variables and absolute numbers and percentages for dichotomous variables. Reproducibility was evaluated with the intraclass correlation coefficient (ICC). For normally distributed continuous variables a paired-samples Student’s t-test and for skewed continuous variables a Wilcoxon test was used to compare RV volumes measured in the LV end-systolic and end-diastolic frame with RV volumes measured in the RV end-systolic and end-diastolic frame. For normally distributed continuous variables an independent Student’s T-test and for skewed continuous variables a Mann-Whitney test was used to compare the difference in RV volumes between normal scans and scans of patients with ToF when measuring RV volumes in the end-systolic and end-diastolic frame of the RV instead of the LV. The relation between QRS duration and change in RV volume and function when using the end-systolic and end-diastolic frame of the RV instead of the LV was analyzed using linear regression. The Statistical Package for the Social Sciences version 16.0 (SPSS Inc, Chicago, IL) was used for all statistical analyses. All statistical tests are two-sided and a P-value of less than .05 was considered statistically significant.

## Results

### Study population

Between January 2008 and January 2011, 65 CMR scans of patients with ToF (50 with complete RBBB, 15 with incomplete RBBB) and 50 normal CMR scans of control subjects were collected. Patients with ToF (37 male, 28 female; median age 28 years, IQR 21 to 37 years) were younger than control subjects (33 male, 17 female; median age 56 years, IQR 41 to 65 years), p<0.001. QRS duration was longer in patients with ToF (145±25 ms) than in control subjects (93±9 ms), p<0.001. Heart rate during the CMR scan was similar in patients with ToF (70±12 bpm) and control subjects (73±15 bpm), p = NS.

### Timing of end-systole and end-diastole

**Figure 1:**
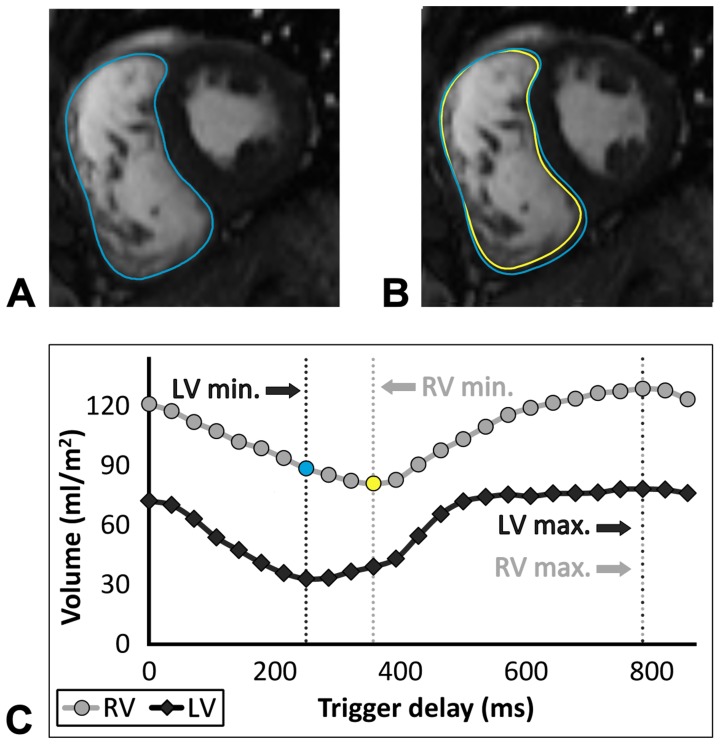
Example of the left and right end-systolic frame and the corresponding time-volume curve. Two short axis images of the end-systolic frame of the LV (**A**) and RV (**B**), and the corresponding time-volume curve (**C**) in a patient with ToF and a complete RBBB. Timing of the RV end-systolic frame is 106 ms (3 frames) delayed compared to LV end-systolic frame. Measuring the RV end-systolic volume in the LV instead of the RV end-systolic frame results in a difference of 9 ml/m^2^. This is visible in the short-axis image of the RV end-systolic frame (**B**) in which the larger blue contour corresponds to the RV contour of the LV end-systolic frame (**A**) and the yellow contour to the RV contour of the RV end-systolic frame. Timing of the end-diastolic frame is the same for the RV and LV. LV = left ventricle, Max. = maximum volume, Min. = minimal volume, RBBB = right bundle branch block, RV = right ventricle.

The difference in frame selection of end-systole and end-diastole between the RV and LV is shown in table 1. Figure 1 shows the time-volume curve of the RV and LV of a patient with ToF and a complete RBBB. In almost all patients with ToF and half of control subjects the end-systolic frame of the RV was delayed compared to the LV. The resulting median difference in timing between the end-systolic frame of the RV and LV was larger in patients with ToF (median −53 ms, IQR −73 to −37 ms) than in control subjects (median −11 ms, IQR −32 to 0 ms), p<0.001. Timing of the end-diastolic frame was not different between the RV and LV in most patients with ToF and control subjects. Also, the resulting median difference in timing between the end-diastolic frame of the RV and LV frame was similar in patients with ToF (median 0 ms, IQR 0 to 36 ms) and control subjects (median 0 ms, IQR 0 to 32 ms), p = NS.

**Table 1:** End-systolic and end-diastolic frame selection of the right ventricle compared to the left ventricle.

	End-systole		End-diastole	
	ToF	Control	ToF	Control
RV–LV frame	N (%)	N (%)	N (%)	N (%)
RV 3 frames earlier	0 (0)	0 (0)	2 (3)	0 (0)
RV 2 frames earlier	0 (0)	0 (0)	2 (3)	2 (4)
RV 1 frame earlier	0 (0)	0 (0)	14 (22)	14 (28)
No difference	4 (6)	25 (50)	38 (58)	34 (64)
RV 1 frame later	26 (40)	25 (50)	8 (12)	0 (0)
RV 2 frames later	28 (43)	0 (0)	0 (0)	0 (0)
RV 3 frames later	7 (11)	0 (0)	1 (2)	0 (0)

LV = left ventricle, RV = right ventricle, ToF = tetralogy of Fallot.

### Change in RV volume and function

**Figure 2:**
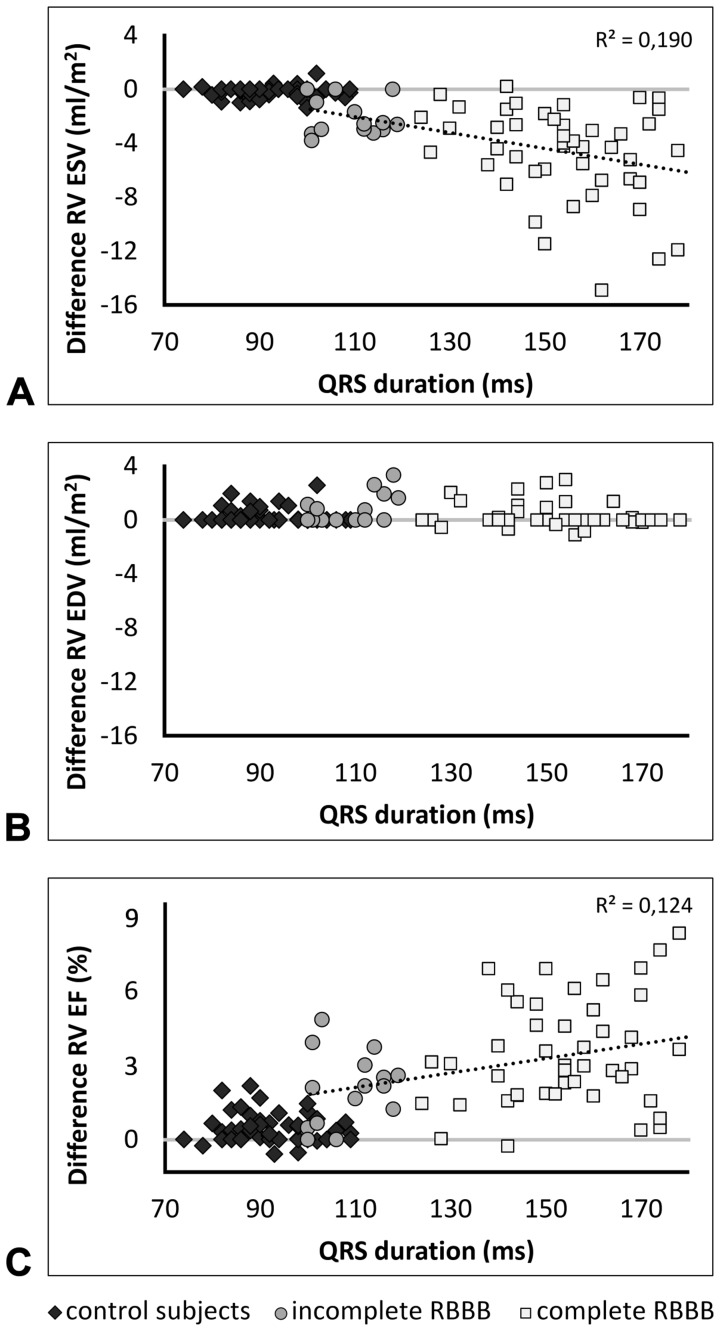
Change in right ventricular volumes and function. Scatterplots of the change in RV end-systolic volume (**A**), end-diastolic volume (**B**) and ejection fraction (**C**) when using the end-systolic and end-diastolic frame of the RV instead of the LV. EDV = end-diastolic volume, EF = ejection fraction, ESV = end-systolic volume, LV = left ventricular, RBBB = right bundle branch block, RV = right ventricular, ToF = tetralogy of Fallot.

Table 2 shows RV volumes and function measured in the end-systolic and end-diastolic frame of the LV and RV. Using the RV end-systolic instead of LV end-systolic frame in patients with ToF, mean RV end-systolic volume was reduced from 78 to 74 ml/m^2^ (p<0.001) while ejection fraction and stroke volume increased from 45 to 48% (p<0.001) and from 62 to 66 ml/m^2^, respectively (p<0.001). ToF patient’s changes in RV end-diastolic volume and the changes in any of these four parameters in the controls were very small, though still significant in paired data analysis. Figure 2 shows the difference in volumes and function when using the end-systolic and end-diastolic frame of the RV instead of the LV. The decrease in RV end-systolic volume was incremental when going from controls to patients with ToF and an incomplete RBBB to patients with ToF and a complete RBBB (p<0.001). In patients with ToF linear regression showed a significant association between QRS duration and change in RV end-systolic volume (B 3.37, CI 1.62–5.13, R^2^ = 0.190, p<0.001), and RV ejection fraction (B 4.25, CI 1.40–7.10, R^2^ = 0.124, p = 0.004) when using the end-systolic and end-diastolic frame of the RV instead of the LV.

**Table 2:** Right ventricular volumes measured in the end-systolic and end-diastolic frame of the left and right ventricle.

	LV frame	RV frame	RV frame–LV frame	
	mean±SD	mean±SD	median (IQR)	*P*
ToF				
RV ESV (ml/m^2^)	77.8±24.1	73.6±23.0	−3.3 (−5.6 to −1.9)	<001
RV EDV (ml/m^2^)	139.6±35.0	140.0±35.0	0.0 (0.0 to 0.9)	<001
RV EF (%)	44.8±7.4	48.0±6.9	2.8 (1.8 to 4.6)	<001
RV SV (ml/m^2^)	61.8±16.4	66.4±16.7	4.1 (2.6 to 5.8)	<001
Control subjects				
RV ESV (ml/m^2^)	35.1±7.6	34.9±7.6	0.0 (−0.4 to 0.0)	003
RV EDV (ml/m^2^)	75.2±12.4	75.4±12.3	0.0 (0.0 to 0.1)	002
RV EF (%)	53.6±4.1	54.0±4.0	0.2 (0.0 to 0.7)	<001
RV SV (ml/m^2^)	40.1±6.1	40.5±6.1	0.2 (0.0 to 0.6)	<001

EDV = end-diastolic volume, EF = ejection fraction, ESV = end-systolic volume, IQR = interquartile range, LV = left ventricle, RV = right ventricle, SD = standard deviation, SV = stroke volume, ToF = tetralogy of Fallot.

The increase of RV ejection fraction is mainly the result of decrease in end-systolic volume when using the end-systolic frame of the RV instead of the LV. Using the end-systolic frame of the RV instead of the LV resulted in a relative increase in ejection fraction of 7%, from 45±7% to 48±7%, in patients with ToF and of 1%, from 54±4% to 54±4%, in control subjects. The relative increase in ejection fraction and stroke volume by using the end-diastolic frame of the RV instead of the LV was <1% in both patients with ToF and control subjects.

In 17 (26%) patients with ToF the absolute increase of ejection fraction exceeded 5% (range 5–8%), figure 2C. In 39 (60%) patients with ToF ejection fraction fell short of the limit of 47% indicating abnormal RV function according to reference values [15]. When using the end-systolic frame of the RV instead of the LV frame, RV function changed to normal in 5 (13%) of these patients. None of the patients with an incomplete RBBB and an abnormal RV function showed improvement to normal values.

### Reproducibility

Intra- and interobserver ICC for RV end-systolic volume, end-diastolic volume and ejection fraction was .98, .91, .87 and .98, .97, .95 in patients with ToF and .94, .93, .88 and .95, .97 and .89 in control subjects, respectively.

## Discussion

Our study is the first to quantitatively demonstrate the difference in RV volumes and function between using the RV and LV end-systolic and end-diastolic frame. Performing cardiac MRI derived measurements of RV volumes using the LV end-systolic and end-diastolic frame, misrepresent RV end-systolic and end-diastolic volumes in many patients with ToF, especially in the frequent case of a complete RBBB. These findings can be of clinical importance in the evaluation of the RV in patients with ToF.

Previous echocardiographic and cardiac MRI studies reported on the electromechanical delay of the RV compared to the LV in patients with congenital heart disease and normal subjects [8]–[10], [17], [18]. The main focus of the echocardiographic studies was to assess intra- and interventricular dyssynchrony and their predictors. They showed that in patients with ToF RV free wall contraction lags behind LV free wall and interventricular septum contraction. Although they did not report on timing of end-systole, it can be expected that end-systole is also delayed. We showed that in most scans of patients with ToF, end-systole of the RV was more than one frame delayed compared to the LV. This was probably largely due to the longer QRS duration in patients with ToF and a complete RBBB. In control subjects the normal physiologically electromechanical delay of the RV could not be detected in every case as the delay was smaller than the time (mean 34±7 ms) between two frames. Therefore, the end-systolic frame of the RV was in the same or one frame later than the end-systolic frame of the LV. In contrast to end-systole, timing of end-diastole of the RV and LV was similar in patients with ToF and control subjects.

One small (N = 12) cardiac MRI study indicated that measuring RV volumes in the two frames preceding RV end-systole causes no clinically significant volume changes despite the observation that end-systole of RV and LV occur in different frames [19]. Our study has made clear that in end-systole the two previous frames have a larger volume. When there is a difference in timing of end-systole of the RV and LV with two or more frames, as is the case in most patients with ToF, this leads to a significant change in volume. When there is a difference in end-systole of the RV and LV in control subjects, this leads to a very small volume change only. QRS duration, unfortunately not documented in the above study [19], appears to be an important parameter as evidenced by the statistically significant correlation with the difference in end-systolic volume when using the end-systolic frame of the RV instead of the LV in patients with ToF obtained in this study. The correlation is weak, however, probably because of our inclusion of a group of patients who are rather homogeneous in terms of QRS duration. In contrast to end-systole, in end-diastole the adjacent frames had a similar volume.

According to the guidelines of the ESC and ACC/AHA, indication for replacement of the pulmonary valve in patients with ToF and moderate/severe pulmonary regurgitation is based on several parameters including RV function [1], [2]. It is important in this context that in 13% of the patients with ToF who were considered to have abnormal function (ejection fraction <47%) [15], ejection fraction increased to normal when using the end-systolic frame of the RV instead of the LV. The change in RV function was mainly due to the decrease in RV end-systolic volume when using the RV frame instead of the LV frame. Studies comparing RV volumes and function before and after pulmonary valve replacement have identified pre-operative threshold values for RV volumes after which volumes can return to normal [3]–[6]. None of these studies describe whether they selected the end-systolic and end-diastolic frame of the RV separately from the LV. The reported threshold for RV end-systolic volume above which RV volume does not return to normal after PVR varies between approximately 80 and 90 ml/m^2^ [3]–[6]. When using a threshold for RV end-systolic volume of >85 ml/m^2^ [3], in our study 25 (39%) patients had volumes above this threshold when measuring RV volumes in the end-systolic frame of the LV. When using the end-systolic frame of the RV instead of the LV, the end-systolic volume dropped below this threshold in 7 (28%) patients. In some of these cases CMR measurements of RV volumes and function may prove to be decisive when considering reoperation. Therefore, RV volumes should be measured in end-systolic of the RV and not of the LV.

### Limitations

Identifying tricuspid valve opening and closing in a 4-chamber or RV 2-chamber view may allow for more accurate selection of the end-systolic frame. However, in the 4-chamber view the opening and closing of the tricuspid valve was not always clearly visible and RV 2-chamber views were not acquired.

There are possible confounders for the difference in timing of end-systole between the studied groups, such as the difference in age, pulmonary stenosis and regurgitation, RV end-systolic and end-diastolic volume and underlying disease. However, it is unlikely that the difference in age will have influenced our results as age does not affect timing of contraction of the right and left ventricle [8]. Possibly, a stronger correlation would have been found between QRS duration and the difference in ejection fraction and end-systolic volume when using the end-systolic and end-diastolic frame of the RV instead of the LV when also patients with ToF and normal QRS duration had been included in this study. To investigate the influence of QRS duration and RBBB more thoroughly, an additional group of patients with ToF and no conduction delays would be useful. However, these patients are rare and in our institution there are only three CMR scans of these patients available over the last three years [1], [20].

Although we have shown that end-systolic volume of the RV in patients with ToF should be measured in the end-systolic frame of the RV instead of the LV, it is uncertain whether this applies to all patients with congenital heart diseases involving the RV and a RBBB.

## Conclusions

Independent selection of the end-systolic and end-diastolic LV and RV frame instead of using the LV end-systolic and end-diastolic frame for RV determinations, results in more accurate end-systolic RV volumes in patients with ToF and a RBBB. The differences are significant and correlate with QRS duration. For clinical decision making in patients with ToF and a RBBB, RV volumes should be measured in the end-systolic frame of the RV instead of the LV.
